# The Effects of Five-Order Nonlinear on the Dynamics of Dark Solitons in Optical Fiber

**DOI:** 10.1155/2013/130734

**Published:** 2013-05-30

**Authors:** Feng-Tao He, Xiao-Lin Wang, Zuo-Liang Duan

**Affiliations:** College of Electronic Engineering, Xi'an University of Post and Telecommunications, Xi'an 710121, China

## Abstract

We study the influence of five-order nonlinear on the dynamic of dark soliton. Starting from the cubic-quintic nonlinear Schrodinger equation with the quadratic phase chirp term, by using a similarity transformation technique, we give the exact solution of dark soliton and calculate the precise expressions of dark soliton's width, amplitude, wave central position, and wave velocity which can describe the dynamic behavior of soliton's evolution. From two different kinds of quadratic phase chirps, we mainly analyze the effect on dark soliton's dynamics which different fiver-order nonlinear term generates. The results show the following two points with quintic nonlinearities coefficient increasing: (1) if the coefficients of the quadratic phase chirp term relate to the propagation distance, the solitary wave displays a periodic change and the soliton's width increases, while its amplitude and wave velocity reduce. (2) If the coefficients of the quadratic phase chirp term do not depend on propagation distance, the wave function only emerges in a fixed area. The soliton's width increases, while its amplitude and the wave velocity reduce.

## 1. Introduction

Optical solitons have been proposed to be used as information carrier for the long-distance optical fiber communications and the optical signal processing. There are two of the most basic physical factors in single mode fiber: group velocity dispersion and self-phase modulation. It arrests pulse broadening resulting from group velocity dispersion, and self-phase modulation causes pulse compression. An optical soliton in fiber is based on the exact balance between the group velocity dispersion and the self-phase modulation. In the ideal situation, propagation of optical solitons in single mode fiber is governed by the famous nonlinear Schrodinger (NLS) equation. Recently, it has been extensively studied theoretically by various methods [1–11]. However, in a real fiber, generally, the core medium is not homogeneous [12]. There is always nonuniformity due to many factors. It is mainly shown in two aspects. One reason is that the variation in the lattice parameters of the fiber medium leading to the distance between two neighboring atoms in the optical fiber is not constant; another reason is that the fiber core diameter fluctuations cause the change of the geometric shape of the fiber. Therefore fiber characteristic parameters such as dispersion, self-phase modulation, and optical fiber loss or gain coefficient are not constants. So this system is described as variable coefficient of the nonlinear schrodinger equation.

The discovery of optical solitons dates back to 1971. Dark solitons form in the normal-dispersion region and appear as an intensity dip whose shape and size do not change. In recent years, the cubic nonlinearities in optical soliton transmission have been attracting more attention, but the general dark solitons under five-order nonlinear term have been much less discussed. When the intensity of the optical pulse propagating inside nonlinear medium exceeds a certain value, it has relatively high coefficient of nonlinear optical materials such as semiconductor doped glass and organic polymer. Even the medium intensity of the optical pulse propagating inside nonlinear medium and the cubic and quintic (CQ) nonlinearities in the governing equation should be taken into consideration [13] because it may affect the spread of the soliton. The research shows that the dark soliton transmission is less affected by environment than bright soliton. Therefore it has potential applications in optical communication system [14].

In this paper, we present the exact solution of dark soliton and calculate the precise expressions of dark soliton's width, amplitude, wave central position, and wave velocity which can describe the dynamic behavior of soliton's evolution. By comparing different quintic nonlinearities coefficients, we analyzed the influence of five-order nonlinear item in soliton transmission.

## 2. Exact Dark Solitons Solution

Recently, the application of (1) with various forms of inhomogeneities has been studied in various papers [15–21]. It should be pointed out that without the residual loss/gain term and five-order nonlinearities term (1) has been studied in different contexts in [15, 16]. With the loss/gain term, (1) has been reported in [19–22] from the light intensity point of view, with five-order nonlinear term being taken into consideration. 

Based on the previous discussions, in this paper we considered a generalized variable coefficients cubic-quintic nonlinear schrodinger (CQNLS) equation. Considering the inhomogeneities in the fiber, the dynamics of the optical pulse propagation are governed by the following inhomogeneous nonlinear schrodinger (INLS) equation:

(1) i∂ψ∂z+β(z)∂2ψ∂τ2+γ(z)|ψ|2ψ+δ(z)|ψ|4ψ  +C(z)τ2ψ+ig(z)ψ=0,

where *ψ*(*z*, *τ*) is the complex envelope of the electrical field in a comoving frame, *z* is the transmission distance, *τ* is the retarded time, *β*(*z*) is the group velocity dispersion parameter, *γ*(*z*) and *δ*(*z*) are the cubic nonlinearity coefficient and the quintic nonlinearity coefficient, respectively; and *C*(*z*) and *g*(*z*) are inhomogeneous parameters related to phase modulation and loss (or gain), which are the functions of the propagation distance *z*. Qian et al. presented without quintic nonlinearities NLS equation of explicit soliton solutions by using the similarity transformations [23]. In this paper, one dark soliton solution has been obtained by the similarity transformation; it can be given by [24]

(2) ψ(z,τ)=aαF0M−g0sinh⁡[p(Z−ωT)]1+Nsinh⁡2[p(Z−ωT)]×exp⁡[iωZ−i(σ+ω22)T]eiϕ(z,τ),

where

(3) $$\begin{array}{c} \sigma =\frac{\left(3N-1\right)p^{2}}{2},\;\;M=\sqrt{\left(3N-2\right)p^{2}N,}\\ p=\sqrt{\frac{3\left(N-1\right)}{2\beta_{0}{\left(3N-2\right)}^{2}}}, \end{array}$$

where *N* is a real number. In order to make the above parameters real, we must define that *N* > 1. Here *ω*, *p*, and *M* are relative to the group velocity, the pulse width, and the amplitude, respectively. *g*_0_  (*g*_0_ < 0) and *F*_0_ are real constants, *a* = *a*(*z*) is arbitrary function of transmission distance, and *α*(*z*) is a positive definite function of transmission distance. The choice of the parameters can affect the dynamics of some solutions, which will be discussed as follows in detail: *Z* = *F*_0_ *α*(*z*)*τ*, *T* = *g*_0_^−1^∫_0_^*z*^ *a*(*z*)*dz* + *T*_0_ is to make (1) integrable and obtain the exact solution, where *T*_0_ is arbitrary real constant. Integrability conditions on (1) for exact solutions by the similarity transformation used in the paper are

(4) β(z)=a2g0α2F02,  δ(z)=G0α2F02g0a,g(z)=αzα(γτγτ+1)−g02βzδG0−γzγ  ,γ(z)=F0α(z)

in which [25–31], *G*_0_ = −*β*_0_ *g*_0_^2^, where *β*_0_ is the arbitrary real constant.

## 3. The Dynamics of Dark Solitons in Optical Fiber

The properties of some solutions have been studied, such as width, amplitude, wave center position, and most of them can be controlled by *a*(*z*), *α*(*z*), and so forth. This situation will be seen apparently in the following by using their exact expressions.

### 3.1. The Coefficients of the Quadratic Phase Chirp Term with Propagation Distance

To study the dynamics of the dark soliton in the optical fiber, we choose *a* = 1, *α* = 1 + *ε*cos⁡(*ω*_0_ *z*), where *α* is arbitrary functions of propagation distance where required, with *ε* ∈ (−1,1), and *ω*_0_ ∈ *R*. Then we can get the coefficients of the quadratic phase chirp term, which is

(5) $$\begin{array}{c} C\left(z\right)=\frac{1}{4}\varepsilon g_{0}\omega_{0}^{2}F_{0}^{2}\left[\varepsilon +2\cos\left(\omega_{0}z\right)+\varepsilon \cos\left(2\omega_{0}z\right)\right]. \end{array}$$

Quintic nonlinearities terms are expressed by

(6) $$\begin{array}{c} \delta \left(z\right)=-\beta_{0}g_{0}{\left[1+\varepsilon \cos\left(\omega_{0}z\right)\right]}^{2}F_{0}^{2}. \end{array}$$

Dark solitary wave intensity is given by

(7) $$\begin{array}{c} {\left|\psi \right|}^{2}=-\frac{M^{2}\sin{\text{h}}^{2}\left[p\left(Z-\omega T\right)\right]}{g_{0}F_{0}\left[1+\varepsilon \cos\left(\omega_{0}z\right)\right]\left[1+N\sin{\text{h}}^{2}\left[p\left(Z-\omega T\right)\right]\right]}, \end{array}$$

where

(8) $$\begin{array}{c} Z=F_{0}\left[1+\varepsilon \cos\left(\omega_{0}z\right)\right]\tau ,\;\;T=g_{0}^{-1}z+T_{0}. \end{array}$$

Thus, the expressions of soliton's wave amplitude, width, wave central position, and wave velocity are written as follows:

(9) |ψ|max⁡2=β0F0M2sinh⁡2(−pωT)[1+εcos⁡(ω0z)]δ(z)[1+Nsinh⁡2(−pωT)],

(10) W(z)=12kln⁡((2+(2+N)sinh⁡2(b)+2sinh⁡(b)    ×2+(1+N)sinh⁡2(b))         ×(2+(2+N)sinh⁡2(b)−2sinh⁡(b)    ×2+(1+N)sinh⁡2(b))−1),

where

(11) k=−δ(z)pβ0g0F0[1+εcos⁡(ω0z)], b=−pωT

(12) xc=−β0g0F0ω[1+εcos⁡(ω0z)][g0−1z+T0]δ(z)

(13) vc=−(β0F0ω[1+εcos⁡(ω0z)]+β0F0ωω0εsin(ω0z)[z+g0T0])×(δ(z))−1

**Figure 1:**
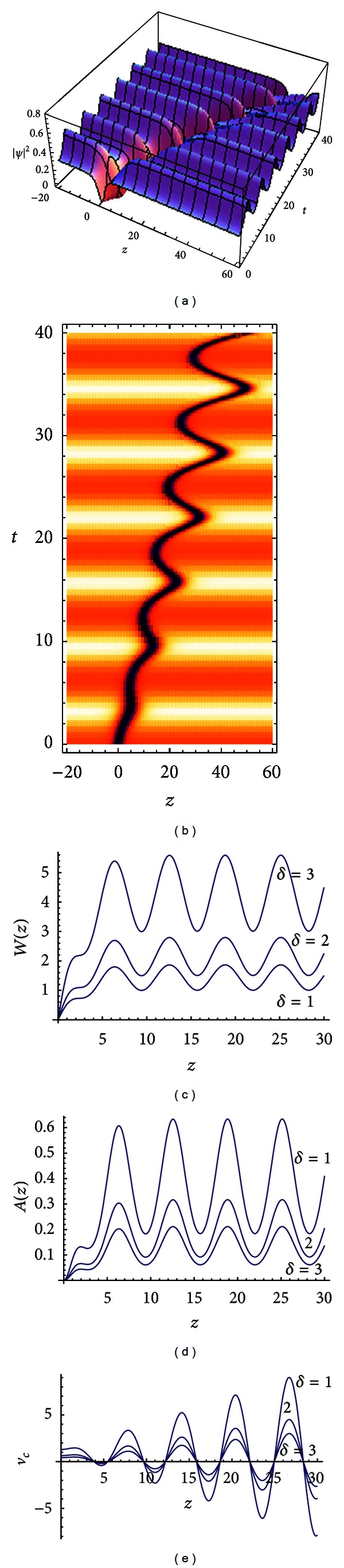
(a) Evolution of the dark solitary wave solution for *ε* = 0.3,  *ω*_0_ = 1. (b) The density plot of (a) with the same parameter. (c) The width of the solution (10). (d) Amplitude of the solution (9). (e) The velocity of the wave center *v*_*c*_ of the soliton (13) with the same parameter. The other parameters are *N* = 2, *β*_0_ = 1, *g*_0_ = −1, *F*_0_ = 1, and *ω* = −1.

Figure 1(a) demonstrates the intensity profiles of the dark soliton wave functions, which vary with time.  Figure 1(b) shows the density in Figure 1(a). Figures 1(c), 1(d), and 1(e) present the change of width, amplitude, and velocity of the wave center through different parameters of quintic nonlinearities *δ* = 1, *δ* = 2, and *δ* = 3, respectively. With the increasing transmission distance, the solitary wave displays a periodic change in the width and amplitude, and the velocity of the wave center executes periodic oscillations and an increase in the magnitude; thus the soliton can spread steadily and have application value in the communication.

From the explicit expressions of (9), (10), (12), and (13), we find that the quintic nonlinearities term *δ*(*z*) affects directly dark soliton's width, amplitude, and wave central position and velocity. With quintic nonlinearities term increasing, the soliton's width increases and its amplitude reduces, while the velocity of the wave center *v*_*c*_ of the soliton also reduces.

### 3.2. The Coefficients of the Quadratic Phase Chirp Term Depend on Propagation Distance

If we take *a* = *α* ^2^,  *C*(*z*) = *λ*, where *λ* is a constant, we can obtain *α* = *C*_0_sech(*C*_0_ *z*), where $C_{0}=\sqrt{-2\lambda \beta_{0}F_{0}/\delta (z)}$. In this case, the coefficients *β*,  *δ* are constants, *γ* is a function of distance, and the gain *g* is vanishing.

Quintic nonlinearities terms are expressed by

(14) $$\begin{array}{c} \delta \left(z\right)=-\beta_{0}g_{0}F_{0}^{2}. \end{array}$$

Dark solitary wave intensity is given by

(15) $$\begin{array}{c} {\left|\psi \right|}^{2}=-\frac{C_{0}M^{2}\text{sech}\left(C_{0}z\right)\sin{\text{h}}^{2}\left[p\left(Z-\omega T\right)\right]}{g_{0}F_{0}\left[1+N\sin{\text{h}}^{2}\left[p\left(Z-\omega T\right)\right]\right]}, \end{array}$$

where

(16) $$\begin{array}{c} Z=F_{0}C_{0}\text{sech}\left(C_{0}z\right)\tau ,\;\;T=g_{0}^{-1}C_{0}\tanh\left(C_{0}z\right)+T_{0}. \end{array}$$

Thus, the expressions of soliton's width, amplitude, wave amplitude, wave central position, and wave velocity are written as follows:

(17) |ψ|max⁡2=β0F0C0M2sech(C0z)sinh2(−pωT)δ(z)[1+Nsinh2(−pωT)],

(18) W(z)=1C0zln⁡2k+4k2−(ln⁡B−2b)22k−4k2−(ln⁡B−2b)2,

where

(19) B=(2+(2+N)sinh2(b)+2sinh⁡(b)    ×2+(1+N)sinh2(b))   ×(2+Nsinh2(b))−1,k=pF0C0,  b=−pωT,

(20) xc=−β0F0ωsinh⁡(C0z)δ(z),

(21) vc=−β0C0F0ωcosh⁡(C0z)δ(z).

**Figure 2:**
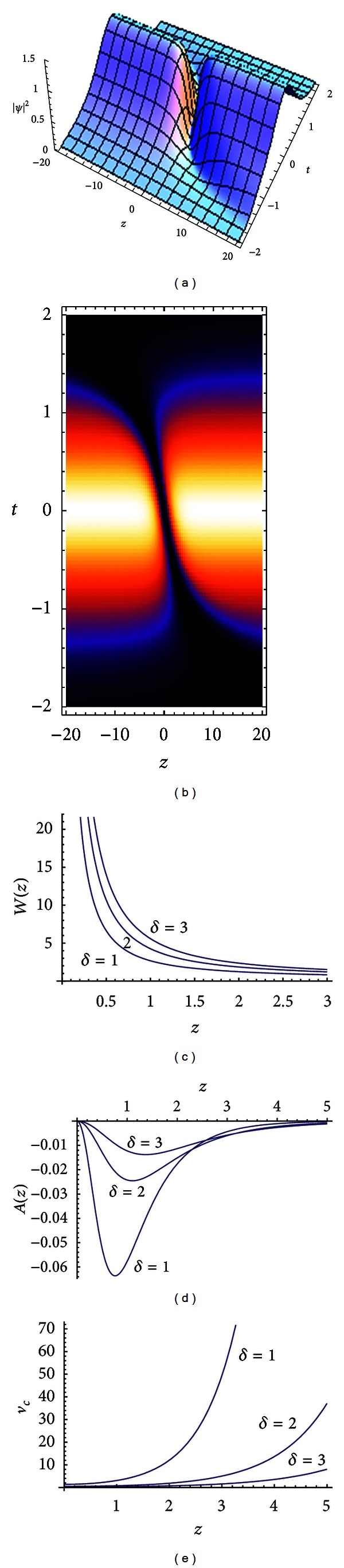
(a) Evolution of the dark solitary wave solution for *λ* = 1. The other parameters are *N* = 2,  *β*_0_ = 1,  *g*_0_ = −1,  *F*_0_ = −1, and *ω* = −1. (b) The density plot of (a) with the same parameter. (c) The width of the solution (18). (d) Amplitude of the solution (17). (e) The velocity of the wave center *v*_*c*_ of the soliton (21) with the same parameter.

In Figure 2, we plot the decaying bent solitary waves to show how they behave as functions of propagation distance.  Figure 2(a) demonstrates the intensity profiles of *ψ*.  Figure 2(b) shows the density of Figure 2(a). Figures 2(c), 2(d), and 2(e) present the change of width, amplitude, and velocity of the wave center through different parameters of quintic nonlinearities *δ* = 1,  *δ* = 2, and *δ* = 3, respectively. We can see from Figures 2(c), 2(d), and 2(e), with the increasing transmission distance, that the solitary wave displays decrease in the width. The amplitude varies from increase to decrease. And the velocity of the wave center increases.

From the explicit expressions of (17), (18), (20), and (21), we find that the quintic nonlinearities term *δ*(*z*) affects directly dark soliton's width, amplitude, wave central position, and wave velocity. With quintic nonlinearities term increasing, the soliton's width increases, and its amplitude reduces, while the velocity of the wave center *v*_*c*_ of the soliton also reduces. We find that the wave function only appears in a fixed area. In other words, the wave function appears to be a local structure; that is, it only emerges within the fixed area, rather than varying with time. Therefore, the structure is a new phenomenon.

## 4. Conclusion

In this paper, we have considered an inhomogeneous nonlinear Schrodinger equation including the five-order nonlinear and chirp term. And by using the similarity transformation, the dark soliton solution has been presented. By changing parameters *a*(*z*), *α*(*z*), and so forth, we have modified the frequency chip. If the coefficients of the quadratic phase chirp term relate to the propagation distance, with the increasing transmission distance, the velocity of the wave center executes periodic oscillations and an increase in the magnitude, and the solitary wave displays a periodic change in the width and amplitude; thus the soliton can spread steadily and have application value in communication. When the coefficients of the quadratic phase chirp term are constants, the wave function appears to be a local structure; that is, it only emerges in the fixed area, rather than varying with time. Therefore, the structure is a new phenomenon. By comparing with different higher order term, we analyzed the influence of five-order nonlinear item on soliton transmission. The results show the main characteristics of the train of optical solitons. So the study of dark solitons in real optical fiber is meaningful. Relevant application deserves to be further studied.

## References

[1] Zakharov VE, Shabat AB (1972). Exact theory of two-dimensional self-focusing and one-dimensional self-modulation of waves in nonlinear media. *Soviet Physics*.

[2] Zakharov VE, Shabat AB (1973). Interaction between solitons in a stable medium. *Soviet Physics*.

[3] Kawata T, Inoue H (1978). Inverse scattering method for the nonlinear evolution equations under nonvanishing conditions. *Journal of the Physical Society of Japan*.

[4] Ma YC (1979). The perturbed plane-wave solutions of the cubic Schrödinger equation. *Studies in Applied Mathematics*.

[5] Hasegawa A, Kodama Y (1982). Amplification and reshaping of optical solitons in a glass fiber—I. *Optics Letters*.

[6] Akhmediev NN, Eleonskii VM, Kulagin NE (1987). Exact first-order solutions of the nonlinear Schrödinger equation. *Theoretical and Mathematical Physics*.

[7] Adachihara H, McLaughlin DW, Moloney JV, Newell AC (1988). Solitary waves as fixed points of infinite-dimensional maps for an optical bistable ring cavity: analysis. *Journal of Mathematical Physics*.

[8] Akhmediev NN, Abnitz SW (1992). Phase detecting of solitons by mixing with a continuous-wave background in an optical fiber. *Journal of the Optical Society of America*.

[9] Elanger NB, Belanger PA (1996). Bright solitons on a cw background. *Optics Communications*.

[10] Hasegawa A, Kodama Y (1995). *Solitons in Optical Communications*.

[11] Agrawal GP (1995). *Nonlinear Fiber Optics*.

[12] Agrawal GP (1995). *Nonlinear Fiber Optics*.

[13] Pushkarov D, Tanev S (1996). Bright and dark solitary wave propagation and bistability in the anomalous dispersion region of optical waveguides with third-and fifth-order nonlinearities. *Optics Communications*.

[14] Agrawal GP (1995). *Nonlinear Fiber Optics*.

[15] Moores JD (1996). Nonlinear compression of chirped solitary waves withand without phase modulation. *Optics Letters*.

[16] Kumar S, Hasegawa A (1997). Quasi-soliton propagation in dispersion-managed optical fibers. *Optics Letters*.

[17] Clarkson PA (1988). Painlevé analysis of the damped, driven nonlinear Schrödinger equation. *Proceedings of the Royal Society of Edinburgh A*.

[18] Burstev SP, Mikhailov AV, Zakharov VE (1987). Inverse scattering method with variable spectral parameter. *Theoretical and Mathematical Physics*.

[19] Vinoj MN, Kuriakose VC, Porsezian K (2001). Optical soliton with damping and frequency chirping in fibre media. *Chaos, Solitons and Fractals*.

[20] Nakkeeran K (2001). An exact soliton solution for an averaged dispersion-managed fibre system equation. *Journal of Physics A*.

[21] Nakkeeran K (2000). Exact soliton solutions for a family of N coupled nonlinear Schrödinger equations in optical fiber media. *Physical Review E*.

[22] Ganapathy R, Kuriakose VC (2003). Soliton interaction in a dispersion-decreasing fiber with effective gain and effective phase modulation. *Chaos, Solitons and Fractals*.

[23] Qian C, Wang LL, Zhang J-F (2011). Solitons of nonlinear Schrödinger equation withvariable-coefficients and interaction. *Acta Physica Sinica*.

[24] He JR, Li HM (2011). Analytical solitary-wave solutions of the generalized nonautonomous cubic-quintic nonlinear Schrödinger equation with different external potentials. *Physical Review E*.

[25] Pushkarov IKh, Pushkarov DI, Tomov IV (1979). Self-action of light beams in nonlinear media: soliton solutions. *Optical and Quantum Electronics*.

[26] Pushkarov IKh, Pushkarov DI (1980). Soliton solutions in some non-linear Schrödinger-like equations. *Reports on Mathematical Physics*.

[27] Pushkarov DI, Tanev S (1996). Bright and dark solitary wave propagation and bistability in the anomalous dispersion region of optical waveguides with third- and fifth-order nonlinearities. *Optics Communications*.

[28] Tanev S, Pushkarov DI (1997). Solitary wave propagation and bistability in the normal dispersion region of highly nonlinear optical fibres and waveguides. *Optics Communications*.

[29] Honzatko P (1996). Interaction of two quasi-solitons in semiconductor-doped optical fibres. *Optics Communications*.

[30] Akhmediev N, Ankiewicz A *Solitons: Nonlinear Pulses and Beams*.

[31] Hao RY, Li L, Li ZH, Yang RC, Zhou GS (2005). A new way to exact quasi-soliton solutions and soliton interaction for the cubic-quintic nonlinear Schrödinger equation with variable coefficients. *Optics Communications*.

